# Oleuropein-driven reprogramming of the myeloid cell compartment to sensitise tumours to PD-1/PD-L1 blockade strategies

**DOI:** 10.1038/s41416-023-02561-y

**Published:** 2024-01-09

**Authors:** Ester Blanco, Noelia Silva-Pilipich, Ana Bocanegra, Luisa Chocarro, Antonio Procopio, Karina Ausín, Joaquín Fernandez-Irigoyen, Leticia Fernández, Nerea Razquin, Ana Igea, Maider Garnica, Miriam Echaide, Hugo Arasanz, Ruth Vera, David Escors, Cristian Smerdou, Grazyna Kochan

**Affiliations:** 1 Oncoimmunology Unit, Navarrabiomed, Fundación Miguel Servet, Universidad Pública de Navarra (UPNA), Hospital Universitario de Navarra (HUN), Instituto de Investigación Sanitaria de Navarra (IdiSNA), Pamplona, Spain; 2 Division of Gene Therapy and Regulation of Gene Expression, Cima Universidad de Navarra, Cancer Center Clínica Universidad Navarra (CCUN), and Instituto de Investigación Sanitaria de Navarra (IdISNA), Pamplona, Spain; 3 Department of Health Sciences, University Magna Graecia of Catanzaro, 88100 Catanzaro, Italy; 4 Proteored-ISCIII, Proteomics Platform, Navarrabiomed, Complejo Hospitalario de Navarra (CHN), Universidad Pública de Navarra (UPNA), IdISNA, Irunlarrea 3, 31008 Pamplona, Spain; 5 Department of Gene Therapy and Regulation of Gene Expression, Center for Applied Medical Research (CIMA), University of Navarra (UNAV), Pamplona, Spain; 6 Medical Oncology Unit, Hospital Universitario de Navarra (HUN), Instituto de Investigación Sanitaria de Navarra (IdiSNA), Pamplona, Spain

**Keywords:** Molecular medicine, Cancer immunotherapy

## Abstract

**Background:**

Previous studies have shown that functional systemic immunity is required for the efficacy of PD-1/PD-L1 blockade immunotherapies in cancer. Hence, systemic reprogramming of immunosuppressive dysfunctional myeloid cells could overcome resistance to cancer immunotherapy.

**Methods:**

Reprogramming of tumour-associated myeloid cells with oleuropein was studied by quantitative differential proteomics, phenotypic and functional assays in mice and lung cancer patients. Combinations of oleuropein and two different delivery methods of anti-PD-1 antibodies were tested in colorectal cancer tumour models and in immunotherapy-resistant lung cancer models.

**Results:**

Oleuropein treatment reprogrammed monocytic and granulocytic myeloid-derived suppressor cells, and tumour-associated macrophages towards differentiation of immunostimulatory subsets. Oleuropein regulated major differentiation programmes associated to immune modulation in myeloid cells, which potentiated T cell responses and PD-1 blockade. PD-1 antibodies were delivered by two different strategies, either systemically or expressed within tumours using a self-amplifying RNA vector. Combination anti-PD-1 therapies with oleuropein increased tumour infiltration by immunostimulatory dendritic cells in draining lymph nodes, leading to systemic antitumour T cell responses. Potent therapeutic activities were achieved in colon cancer and lung cancer models resistant to immunotherapies, even leading to complete tumour regression.

**Discussion:**

Oleuropein significantly improves the outcome of PD-1/PD-L1 blockade immunotherapy strategies by reprogramming myeloid cells.

## Background

Tumour-associated myeloid cells (TAMCs) are major promoters of tumour progression and metastasis, of which myeloid-derived suppressor cells (MDSCs) and tumour-associated macrophages (TAMs) are the main contributors to cancer-associated immunosuppression [1]. MDSCs are classified into two types according to their phenotype: monocytic (m-MDSCs) and granulocytic (g-MDSCs) [1]. Elevation of TAMC numbers systemically and within the tumour microenvironment is associated with resistance to chemotherapy, targeted therapy, and immunotherapy [2, 3]. Overall, an increase in the number of these populations is a poor prognostic factor [4, 5]. In fact, these myeloid subsets have also been used as biomarkers for clinical responses to immune checkpoint blockade with monoclonal antibodies (mAbs) against programmed death 1 (PD‐1) or its ligand (PD‐L1) [3, 6–8]. Previous studies have shown that reprogramming of immunosuppressive myeloid cells towards immunostimulatory subsets does take place in responder patients under PD-1/PD-L1 blockade immunotherapies [3, 6, 9, 10]. Therefore, TAMC reprogramming before immune checkpoint blockade could overcome resistance to therapy by potentiating the “immunogenic metabolism” which controls key processes such as phagocytosis and antigen presentation [11, 12]. Metabolic pathways are important regulators of myeloid cell phenotype and function [13, 14]. We previously identified key metabolic networks in mouse MDSCs and TAMs regulating their activities [15], using ex vivo-differentiated myeloid cells modelling tumour-associated subsets [9, 16, 17]. Glycolytic pathways, oxidative phosphorylation, and lipid metabolism were activated in immunosuppressive myeloid cells. The main differential characteristics among murine TAMCs subsets were also identified. These included pathways related to fatty acid oxidation, which are emerging as promising therapeutic targets [15, 18]. Some modulators of fatty acid and lipid metabolic pathways have been shown to reprogramme immunity in cancer [18–20], including oleuropein, an olive oil bisphenol derivative used to regulate chronic inflammation and oxidation. Oleuropein demonstrated anti-proliferative capacities over cancer cells in vitro and in vivo [21–23]. However, the reprogramming capacities of oleuropein over TAMCs and its capacity to potentiate PD-1/PD-L1 blockade have not been evaluated yet.

Current PD-1/PD-L1 blockade strategies are based on systemic administration of the therapeutic mAbs once or twice a month. However, this mode of delivery is characterised by poor antibody penetration into solid tumours. Moreover, anti-PD-1/anti-PD-L1 antibodies can cause inflammatory adverse events and even hyperprogressive disease in immunotherapy-treated patients [24]. Local delivery of mAbs within tumours could circumvent these problems by increasing the therapeutic index while reducing off-target toxicities [25–27]. PD-1/PD-L1 blockade antibodies have been delivered into tumours using cytotoxic viral vectors to increase immunogenicity [28, 29]. For example, RNA vectors based on Semliki Forest virus (SFV) have been used to deliver transgenes into target cells. Short local expression of anti-PD-L1 mAb or nanobodies with SFV vectors demonstrated significant therapeutic efficacy in colon adenocarcinoma and melanoma models [30, 31]. Infection of tumour cells also caused apoptosis and the induction of potent type I interferon responses which further potentiated antitumour responses.

Here, we conducted an in-depth characterisation of the reprogramming capacities of oleuropein over cancer-associated myeloid cells to overcome the resistance to PD-1/PD-L1 blockade immunotherapies associated to these immune cell types. Oleuropein-regulated interactome networks were identified by differential quantitative proteomics in myeloid cell subsets which were associated to reprogramming towards immune stimulation. Reprogramming was confirmed phenotypically and functionally. Oleuropein was combined with two PD-1 blockade strategies, either systemically or locally by delivering anti-PD-1 mAb with a self-amplifying SFV RNA vector. Oleuropein combined with PD-1 blockade showed significant therapeutic activities in cancer models resistant to PD-1 blockade.

## Methods

### Animal studies

Four-week-old female C57BL/6 and BALB/c female mice were purchased from Envigo (Barcelona, Spain). Animal studies were approved by the University of Navarra ethics committee (E20-22(078-19E1) and 077-19). ARRIVE reporting guidelines were followed.

### Cells

BHK-21 cells (ATCC-CCL10) were grown as described [31]. LLC cells were grown in DMEM (GIBCO BRL, UK) supplemented with 10% FBS, 2 mM glutamine, and antibiotics. LLC cells expressing mouse GM-CSF (LLC-GMCSF) were generated by lentivector transduction following published methodologies [9, 15–17]. MC38 cells were provided by Dr. Karl E. Hellström (University of Washington, Seattle, WA). These cells were cultured in RPMI-1640 medium (Lonza, Switzerland) supplemented with 10% FBS, 2 mM glutamine, 20 mM HEPES, antibiotics and 50 µM 2-mercaptoethanol. H1299 cells (ATCC- CRL5803) and A549 cells (ATCC-CCL-185) were purchased from the ATCC and cultured in RPMI 1640 Medium with L-Glutamine (Lonza) supplemented with 5% fetal bovine serum (FBS) and 5% Penicillin-Streptomycin 10,000 U/ml (GIBCO BRL, UK). All cell lines were tested once a month for mycoplasma by PCR.

### Ex-vivo differentiation and purification of murine MDSCs and TAMs

Granulocytic-MDSCs, monocytic-MDSCs and TAMs were differentiated from mouse bone marrow following published procedures using conditioning medium produced by LLC tumour cells stably expressing GMCSF and MCSF cells [9, 15–17, 32]. MDSC subsets were purified using the myeloid-derived suppressor cell isolation kit (Miltenyi Biotec, Bergisch, Germany). MDSCs and TAMs were seeded with 50 µM oleuropein and incubated for 7 days. Oleuropein was obtained as described in [33, 34].

### Mixed lymphocyte reaction (MLR)

MLR were carried out following standard procedures [16]. Briefly, 100,000 MDSCs or TAMs from C57BL/6 mice were co-cultured in 96-well plates with 100,000 lymphocytes from BALB/c mice. Co-cultured cells were treated with 50 µM oleuropein and compared with control vehicle. After 5 days, T-lymphocyte activation, differentiation and proliferation were assessed by flow cytometry. T cell activation and proliferation markers (Ki-67) and pro-inflammatory cytokine production (IL-2 and IFNγ) were evaluated by flow cytometry as described [35].

### Mass spectrometry-based quantitative (shotgun) proteomics and bioinformatics analysis

Five biological replicates per sample were analysed (m-MDSC, g-MDSC and TAM). Cell pellets were homogenised in lysis buffer (7 M urea, 2 M thiourea 50 mM DTT). Protein extracts were diluted in Laemmli buffer and loaded into a 0.75 mm thick polyacrylamide gel with a 4% stacking gel casted over a 12.5% resolving gel. Proteomes were concentrated in the stacking/resolving interface. Bands were stained with Coomassie Brilliant Blue, excised and cleaved with trypsin (Promega, WI, USA; 1:20, w/w) at 37 °C for 16 h as previously described [36]. Peptide purification and concentration was performed using C18 Zip Tip Solid Phase Extraction (Millipore).

### LC-MS/MS

Peptides were separated by reverse phase chromatography using an UltiMate 3000 UHLPC System (Thermo Scientific, MA, USA) fitted with an Aurora packed emitter column (Ionopticks, 25 cm × 75 µm ID, 1.6 µm C18). Samples were desalted and concentrated into an Acclaim PepMap column (ThermoFisher, 0,5 cm × 300 µm ID, 5 µm C18). Mobile phases were 100% water 0.1% formic acid (FA) (buffer A) and 100% Acetonitrile 0.1% FA (buffer B). Column gradient was developed in a 120 min two-step gradient from 5% B to 20% B in 90 min and 20% B to 32% B in 30 min. Column was equilibrated in 95% B for 10 min and 5% B for 20 min. The precolumn was in line with the column and the flow maintained all along the gradient at 300 nl/min. Temperature was maintained at 40 °C and interfaced online with the Orbitrap Exploris 480 MS. Spray voltage were set to 2 kV, funnel RF level at 40, and heated capillary temperature at 300 °C. Full MS resolutions were set to 1,200,000 at *m*/*z* 200 and full MS Automatic gain control (ACG) target was set to Standard with an IT mode Auto. Mass range was set to 375–1500. AGC target value for fragment spectra was set to Standard with a resolution of 15,000 and 3 s for cycle time. Intensity threshold was kept at 8E3. Isolation width was set at 1.4 *m*/*z*. Normalised collision energy was set at 30%. Data were acquired in centroid mode using positive polarity and peptide match was set to off, and isotope exclusion was on.

### Data analysis

Raw files were processed with MaxQuant [37] v1.6.17.0 using the integrated Andromeda Search engine [38]. All data were searched against a target/decoy version of the mouse Uniprot Reference Proteome with March 2021 release. First search peptide tolerance was set to 20 ppm, main search peptide tolerance was set to 4.5 ppm. Fragment mass tolerance was set to 20 ppm. Trypsin was specified as enzyme, cleaving after carbamidomethylation of cysteine was specified as fixed modification and peptide N-terminal acetylation, oxidation of methionine, deamidation of asparagine and glutamine and pyro-glutamate formation from glutamine and glutamate were considered variable modifications with a total of two variable modifications per peptide. “Maximum peptide mass” were set to 7500 Da, the “modified peptide minimum score” and “unmodified peptide minimum score” were set to 25 and everything else was set to the default values, including the false discovery rate limit of 1% on both the peptide and protein levels. The Perseus software (version 1.6.14.0) [39] was used for statistical analysis and data visualisation. Metascape [40] was used to identify enrichment GO processes, KEGG pathways, reactome gene sets and canonical pathways in our proteomes. For multicomparisons, ANOVA tests were performed followed by pairwise comparisons Student’s *t* tests. Data were filtered according to −Log *p* value *x* ≥ 1.3 and; log2 fold change *x* ≥ 0.38 and *x* ≤ −0.38. Construction of functional interactomes from up- or down-regulated proteins was carried out with the Ingenuity Pathway Analysis Tool (Qiagen) (https://www.qiagen.com/us/products/discovery-and-translational-research/next-generation-sequencing/informatics-and-data/interpretation-content-databases/ingenuity-pathway-analysis/).

### Real-time cell analysis

Cytotoxicity in cell cultures was evaluated by xCELLigence Real-Time Cell Analysis (RTCA) (Roche Diagnostics GmbH, Mannheim, Germany) as described before [3, 17]. Briefly, LLC, H1299, and A549 cells were seeded at a density of 3 × 10^3^ cells/well on gold microelectrode-embedded 16-well microplates (E-plates; Roche Diagnostics, Basel, Switzerland) and incubated at 37 °C with 5% CO_2_. Impedance was recorded at 15 min intervals. Oleuropein (25–250 µM) was added to the culture at seeding time. Incubations were performed in a volume of 100 µl up to 120 h. Delta Cell Index values were evaluated with RTCA-DP software (Roche Diagnostics GmbH). Delta CI (Delta Cell index) was used to normalise data.

### Cell staining and flow cytometry

Maleimide dye was used (Promokine) to discriminate living from dead cells. Surface and intracellular staining were carried out as described [15, 35] with fluorochrome-conjugated antibody clones: AF488 anti-CD49d (R1-2), PE anti-CD4 (GK1.5), APCanti-CD8(53-6.7), brilliant Violet 510™ anti-I-A/I-E (M5/114.15.2,PE/Cyanine7anti-Ly-6G (1A8), and APC anti-CD80 (16-10A1) (Biolegend, CA, USA), FITC anti-IL-12 (C15.6) and BV421 anti-IL-2 (JES6-5H4) (BD Bioscience, NJ, USA), APC-Vio ® 770 anti-Ly-6C (REA796), PE REAfinity™ anti-F4/80 (REA126), and APC anti-CD68 (REA886) (Miltenyi), PerCP-Cyanine5.5 Anti-Human/Mouse CD11b (M1/70) (Tonbo, CA, USA), and AF488 anti-IFNγ (Clone XMG1.2) (BD Pharmigen, NJ, USA). For human cells, we used the following fluorochrome-conjugated antibodies: PerCP-Cy5.5-anti-CD11b (integrin alfa M) (M1/70), Violet Fluor 450 anti-CD14 antibody (61D3) (Tonbo), PE anti-CD115 (CSF-1R) (9-4D2-1E4) (Biolegend), PE-Cy™7 anti-CD11c (Clone B-ly6) (BD Bioscience), and APC anti-HLA-DR (REA517) (Miltenyi).

Infiltrating immune cells were studied with the fluorochrome-conjugated antibody clones: FAPCH7 anti-CD45 (S18009F), BV605 anti-F4/80 (BM8), AF700 anti-CD11c(N418), BV650 anti-CD14 (Sa14-2), PE anti-CD115 (9-4D2-1E4), BV785 anti-PD-L1 (29E.2A3), Alexa Fluor® 488 anti-CD49d (R1-2), BV510 anti-Ly6c (HK1.4), BV421 anti-VISTA (MH5A), BV605 anti-CD3 (17A2), AF700 anti-GrzB (QA16A02), BV650 anti-NKp46 (29A1.4), BV785 anti-CD4 (GK1.5), PE/Cyanine7 anti-Ly-6G (1A8), APC anti-CD137 (17B5), BV510 anti-CD8 (53-6.7), PE anti-TIM3(RMT3-23) and BV421 anti-LAG3 (C9B7W) (Biolegend), FITC anti-PD-1 (J43) (BD Bioscience), PerCP-Cy5.5-CD11b (integrin alfa M) (M1/70) (Tonbo), APC anti-CD68 (REA886) (Miltenyi). Data were collected using the FACSCanto Flow Cytometer (BD Biosciences) for ex vivo and in vitro experiments at Navarrabiomed Biomedical Research Center and Cyroflex LX flow cytometer (Beckman Counter) to characterise tumour and lymph nodes at CIMA Universidad de Navarra. Data were analysed with Flowjo.

### Myeloid cell samples from non-small cell lung cancer (NSCLC) patients

Twenty one patients with locally advanced or metastatic NSCLC treated with the immune checkpoint inhibitor (ICI) pembrolizumab (anti-PD-1) were recruited for the study. The characteristics of the cohort under study are described in [3, 35]. This observational study was approved by the Ethics Committee of Clinical Investigations at the University Hospital of Navarre (reference number: PI_2020/115). Blood samples from non-small cell lung cancer (NSCLC) patients were collected prior to treatment and before administration of each immunotherapy cycle, after a signed informed consent from the patients.

PBMCs were isolated and grown in vitro for 24 h in TEXCMACs medium (Miltenyi Biotec) as described [3, 35]. Myeloid cells were obtained by adherence to plastic for 24 h. Then, 50,000 myeloid cells were seeded per well with 50 µM oleuropein in 96-well plates and incubated for seven days.

### Production and characterisation of recombinant SFV-αPD1 vector

The SFV-αPD1 vector was engineered by cloning the coding sequence of an anti-PD-1 mAb synthesised by GenScript [31]. This gene contains the sequences corresponding to the heavy (IgG1 isotype) and light (lambda) chains of an aPD-1 mAb fused by the foot and mouth disease virus 2A self-cleaving peptide sequence preceded by a furin cleavage sequence. The gene was subcloned into Apa I site in the SFV-b12A vector following the same strategy used to construct SFV-αPDL1, which encodes a mAb against PD-L1 [30]. SFV-LacZ was described previously [41].

SFV-αPD1 viral particles (VPs) were titrated in SFV-infected BHK-21 cells following published procedures [41]. Vector titre were approximately 6 × 10^10^ VPs/ml for SFV-αPD1 and 2 × 10^10^ VPs/ml for SFV-LacZ.

Anti-PD1 mAb expression was corroborated in vitro in infected BHK-21 and LLC cell monolayers with SFV VPs at a multiplicity of infection (MOI) of 20. After 24 h, anti-PD1 mAb expression was quantified in cell extracts and supernatants by ELISA and Western blot. Expressed recombinant anti-PD1 mAb in supernatants or lysates was quantified by ELISA (Mabtech, Sweden) following the manufacturer’s instructions. A commercial IgG1 mAb was used as a standard curve (Santa Cruz Biotechnology).

The binding capacity of the anti-PD1 mAb was quantified by a specific PD-1 binding ELISA developed by our laboratory. Briefly, ELISA plates were coated with 1 µg/ml recombinant murine PD-1 fused to human IgG1 Fc (R&D, Minneapolis, USA) and incubated overnight at 4 °C. Subsequently, the plates were incubated with serial dilutions of the samples. Finally, the wells were incubated with a goat polyclonal anti-mouse IgG1 conjugated with peroxidase (Abcam), and the substrate tetramethylbenzidine (TMB) was used to develop the reaction and stopped with 2 M NaOH. The absorbance was measured in an ELISA reader at 450 nm.

To confirm the blockade capacities of the expressed anti-PD1 mAb, a competitive inhibition ELISA was developed. Briefly, ELISA plates were coated with 0.025 μg/ml PD-L1 fused to human IgG1 Fc (R&D). Then, wells were incubated with a mixture of 0.3 μg/ml biotinylated PD-1 (BPS Bioscience) and different concentrations of the recombinant anti-PD-1 mAb. Finally, wells were incubated with streptavidin-peroxidase and the reaction was stopped and read. As a positive control, purified anti-PD-1 mAb was used (Bioscience).

### Mouse tumour models and therapies

Sample sizes were calculated to achieve a minimum power of 0.8 for *F*-based tests taking into consideration a large effect size (*f* = 04). Power calculations were carried out with Gpower 3.1.9.7. Blinding was used for data analysis and correlation with survival. C57BL/6 female mice were randomly allocated and subcutaneously (s.c.) injected with 10^6^ LLC cells or 0.5 × 10^6^ MC38 cells per animal. No blinding was established for the experiments. When tumour growth reached an average diameter of 3.5 mm (day 0), 10^9^ VPs of SFV vectors were administered intratumourally (i.t.) in a volume of 50 µl. Control mice received the same volume of saline. Some groups of mice received 100 µg of anti-PD-1 mAb (RPMI-14, BioXCell) intraperitoneally (i.p.) at days 0, 5, and 13. When appropriate, mice received 300 µg oleuropein at days -1, 2, 4, 6, and 8. As negative control, the same volume of saline was injected.

The two perpendicular tumour diameters were measured every two days. The size was calculated using the formula: Size =Length × Width. Mice were humanely sacrificed when tumour size reached ~150 mm^2^, or when tumour ulceration or discomfort were observed. For rechallenge experiments in the MC38 model, mice that rejected tumours were injected s.c. with 5 × 10^5^ MC38 cells in the left flank 3 months after the first tumour inoculation. Naïve mice were included as controls. Tumour growth was monitored for 2 months. Tests for liver function were carried out by measuring serum ALT, AST, and amylase on a Cobas Mira Plus Analyser (c-311 Roche Diagnostics).

For immunophenotyping of tumour immune infiltrates, draining lymph nodes (DLNs) and spleens were extracted from mice harbouring LLC-derived tumours. Tumours, DLNs and spleens were isolated on day 5 post-treatment, and immune infiltrates analysed by flow cytometry. Briefly, tumours were treated with 100 μg/ml LIBERASE TL (5401119001 ROCHE, France) and 100 μg/ml DNase I (ROCHE, France). After mechanical tissue dissociation and incubation at 37 °C for 1 h, cells were filtered through a 70-mm nylon mesh (BD Falcon, BD Bioscience, San Jose, CA, USA), washed with PBS, treated with ammonium-Chloride-Potassium (ACK) lysing buffer (Gibco, ThermoFisher, USA), and washed again with PBS. DNLs and spleens were homogenised in PBS by mechanical tissue dissociation. Cell stainings and flow cytometry were then carried out.

### ELISPOT

ELISPOTs were performed with the IFNγ ELISPOT kit (BD Biosciences) following the instructions of the manufacturer. The number of IFNγ-producing cells was quantified in splenocytes. Briefly, a 96-well plate was coated with an anti-mouse IFN-γ capture antibody at a concentration of 5 μg/ml in PBS. After overnight incubation at 4 °C, the antibody was removed and the wells were blocked with RPMI-1640 complete medium for 2 h at room temperature (RT). Splenocytes (7 × 10^5^/well) were co-cultured with irradiated LLC cells (using 14,000 cGy) at a 10:170 lymphocyte:tumour cell ratio. The following day IFN-γ+ spots were revealed and counted in an automated ELISPOT reader (CTL, Aalen, Germany).

### Statistical analyses

Statistical analyses were performed with the GraphPad Prism 8.3 software package. No data was discarded from analyses. Cytometry data from ex vivo or in vitro experiments were normalised to their respective controls (samples not treated with oleuropein). All in vitro and ex vivo results from cytometry analysis were normalised against their controls (treated sample value- control sample value). Normality was evaluated with Kolmogorov–Smirnov and Shapiro–Wilk tests (in the case of samples with *n* < 10). Homocedasticity was evaluated by the chi-squared test. Statistical analyses were performed with paired-tailed Student’s *t* test (paired dependent *t* test), with a significance level of *p* < 0.05. For non-normally distributed data or with intrinsic variability the Wilcoxon matched pairs test was used. For RTCA analyses, the slope between two points was calculated using the following formula: m = (y1 − y2)/(x1 − x2).

Tumour measurements of in vivo experiments were represented as tumour surface (mm^2^) and plotted either as individual data points for one individual mouse, or as mean ± SD as indicated in figure legends. For in vivo experiments, one-way ANOVA test was used for multicomparisons, followed by a posteriori Tukey’s pair-wise comparisons. When indicated, two-tailed Student’s *t* test was applied to compare two experimental groups. For time-series analyses, data were compared using the extra sum-of-squares *F* test and fitted to second-order polynomial equation. Survival was represented by Kaplan–Meier plots and analysed by log-rank test.

## Results

### Reprogramming of TAMCs by oleuropein

**Fig. 1:**
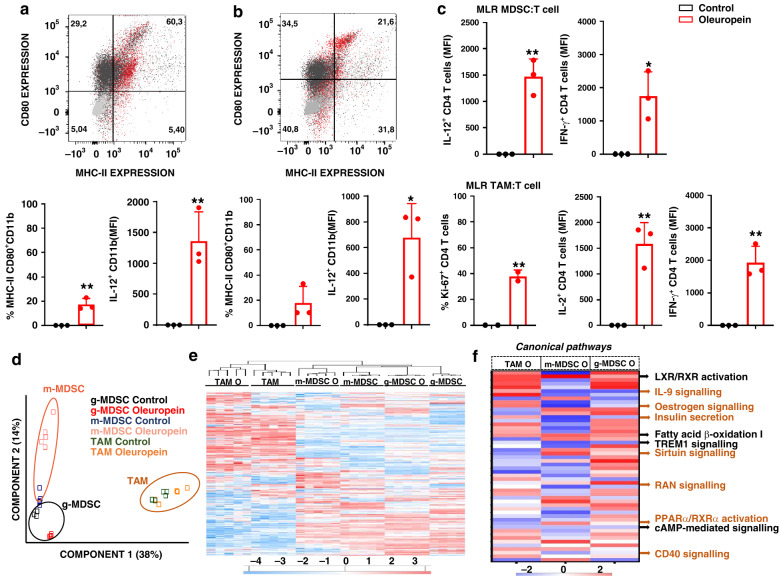
Oleuropein reprogrammes immunosuppressive myeloid cells. **a** Flow cytometry density plots of MHC-II and CD80 expression in MDSCs as indicated. Control MDSC cells in black, oleuropein-treated MDSCs in red and unstained control cells in grey. Percentage of events within each gate are indicated within each quadrant. Bar graphs represent the percentage of CD11b+ MDSCs expressing MHC-II and CD80 (left) and IL12 (right). Percentages and mean fluorescent intensities (MFI) were normalised to those from control cells (*n* = 3 biologically independent cultures). Standard deviations are plotted as error bars. **b** As in (**a**) but with TAMs. **c** Bar graphs represent Ki-67, IL-2 and IFN-γ expression within CD4 cells in MLR using either MDSC as stimulator cells (upper graphs) or TAMs (bottom graphs), treated or untreated with 50 µM oleuropein as indicated. Expressions were normalised to those from control cultures (*n* = 3). Data shown with means and standard deviations are error bars. Statistical significance was tested by two-tailed *t* tests. **d** Principal component analyses (PCA) of proteomic data from the indicated myeloid cell types oleuropein-treated or untreated as indicated in the legend within the graph. PCA is performed with 2642 proteins from 5 independent biological replicates. **e** Hierarchical clustering of differentially expressed proteins (*p* < 0.001) between oleuropein-treated (O) or untreated MDSC and TAM (TAM C) biological replicate cultures (*n* = 5 or *n* = 4). Red and blue, up and down-regulated proteins, respectively. **f** Canonical pathways enriched in m-MDSC, g-MDSCs, and TAMs treated with oleuropein compared with untreated cells. Data are represented as heatmaps of *Z*-score from representative genes of the indicated canonical pathways. Red and blue indicate predicted activation or inhibition, respectively. In black, main canonical pathways regulated in all TAMCs. In brown, main canonical pathways regulated in MDSCs. *p < 0.05; **p < 0.01.

The capacities of oleuropein to overcome the immunomodulatory functions of MDSCs and TAMs were evaluated over MDSCs and TAMs differentiated ex vivo. Oleuropein treatment caused morphological changes in immunosuppressive myeloid cells (Fig. S1a). After treatment, MDSCs acquired the characteristic elongated shapes of dendritic cells (DCs), while TAMs resembled M0 (uncommitted) or M1 (immunostimulatory) macrophages. Importantly, oleuropein upregulated the expression of surface markers of activation and antigen presentation together with significant production of IL-12 (Fig. 1a, b). Mixed lymphocyte reactions (MLR) were used to test T cell activating capacities by oleuropein-treated myeloid cells. Untreated MDSCs and TAMs failed to stimulate T cells. In contrast, these cells potently stimulated CD4 T cell proliferation and production of interferon-gamma (IFN-γ) and IL-2 following oleuropein treatment (Figs. 1c and S1b). We hypothesised that this functional change could be caused by myeloid cell reprogramming towards differentiation of immunostimulatory subsets. To find out if this was the case, the proteomes of oleuropein-treated MDSC and TAMs were compared to untreated controls (Fig. 1d, e). Differentially-regulated proteins were identified with a false discovery rate lower than 1% (912, 2620, and 955 for m-MDSC, g-MDSC, and TAM, respectively) (Fig. S1c). Cluster analyses confirmed significant changes in proteomes of oleuropein-treated TAMCs (Fig. 1e). Ingenuity pathway analysis (IPA) algorithms identified the pathways specifically altered by oleuropein (Fig. 1f). Protein networks regulating inflammation were down-modulated. Pathways and regulators associated to immunosuppressive functions in MDSCs and TAMs were reduced, including decreased expression of the TREM1 receptor, cyclic adenosine monophosphate (cAMP), RAN signalling, IL-9 and CD40 [42, 43]. Additionally, oleuropein treatment up-regulated pathways such as LXR/RXR and fatty acid β-oxidation I signalling (Figs. 1f and S2), associated to MDSC reduction in vivo [44, 45]. The lipid metabolism was altered through increased expression of regulators such as sirtuin, polyamines, and PPAR-α/ RXR-α, while oestrogen and insulin signalling were downregulated (Fig. 1f). Oleuropein altered signalling pathways associated to fatty acid β-oxidation, including a reduction of IL-36γ signalling and Lyz1 (Fig. S2a, c, d, f). The expression of some proteins was specific for particular myeloid subsets. For example, Ftap4, Acsf2 and Ilrn1 were inhibited in m-MDSC but not in g-MDSC (Fig. S2c, e). Glut1, a regulator of glucose uptake which drives M1 polarisation [46], was elevated in TAMs treated with oleuropein (Fig. S2f). Summarising, oleuropein caused major global reprogramming in murine myeloid cell subsets by deactivating immunosuppressive pathways.

**Fig. 2:**
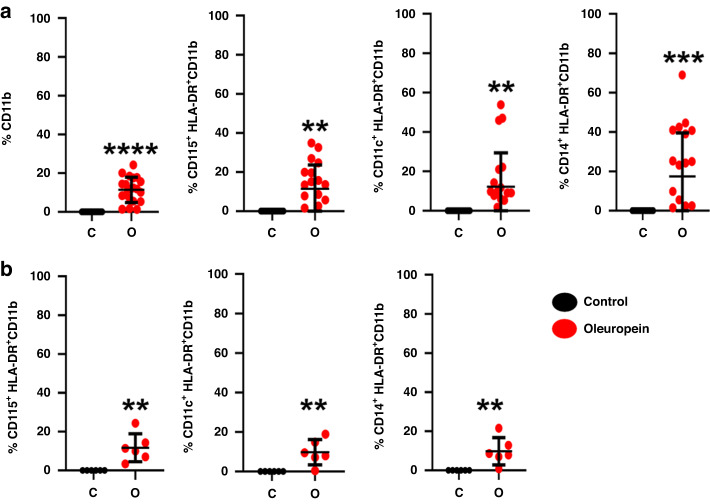
Oleuropein leads to differentiation of immunogenic myeloid cell types from NSCLC patients and healthy donors. Graphs represent the percentage of the indicated myeloid cell phenotypes from NSCL patients (**a**) and healthy donors (**b**) after incubation with 50 µM oleuropein compared to control non-treated cells as indicated. The percentages of myeloid cells that express the indicated markers have been normalised to those from control cultures (*n* = 21 and 6 independent replicate samples for NSCLC patients and healthy donors, respectively). Individual data points, means and error bars (standard deviations) are shown. Statistical significance was tested by Student’s *t* test. Wilcoxon test was used for healthy controls. **p < 0.01), ***p < 0.001; ****p < 0.0001.

We tested whether similar results could be obtained in human myeloid cells isolated from peripheral blood of NSCLC patients. Following oleuropein treatment, an elevation of CD11b+ cells was observed with an increase in the percentage of myeloid cells expressing CD115 HLA-DR, CD14 HLA-DR, and CD11c HLA-DR (Fig. 2a). Hence, oleuropein caused a diversification towards monocytic-like and dendritic cell (DC)-like phenotypes. These results were not limited to myeloid cells from cancer patients, as similar results were obtained with myeloid cells from healthy donors (Fig. 2b).

### Oleuropein sensitises a lung cancer model resistant to PD-1 blockade and potentiates immunotherapy

The capacities of oleuropein to inhibit the growth of lung adenocarcinoma cells were studied in cultures of mouse LCC and human A549 and H1299 cell lines. Oleuropein retarded their growth within a concentration range of 50–250 μM (Fig. S3a, c). To test antitumour capacities in vivo, LLC cells were implanted subcutaneously in mice and tumours were allowed to grow to an average diameter of around 12 mm^2^. Tumours derived from LCC cells are poorly immunogenic and notoriously refractory to PD-1/PD-L1 blockade [3, 9, 47, 48]. Oleuropein was then administered intraperitoneally every other day for 8 days (Fig. S4a). Oleuropein significantly delayed tumour growth and increased survival (Fig. S4b, c), with most of the mice responding to the treatment (Fig. S4d).

**Fig. 3:**
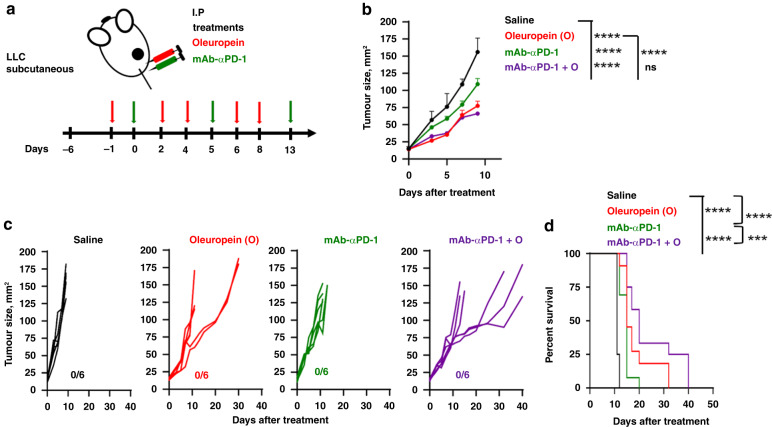
Oleuropein in combination with systemic delivery of anti-PD-1 mAb in LLC tumours. **a** Schematic diagram of the experimental in vivo model. One million LLC cells were inoculated subcutaneously into the right flank of C57BL/6 mice. Five days later (day 0), when the average diameter was 3–4 mm, animals received 100 µg of anti-PD-1 mAb (mAb-αPD1), given i.p. at days 0, 5 and 12, alone or in combination with oleuropein (O) (300 µg), which was injected i.p. at days −1, 2, 4, 6, and 8. The combination group received both compounds in the same dosage as individual groups (mAb-αPD1 + O). The control group received the same volume of saline. **b** Graph represents tumour growth, as mean tumour size (mm^2^) + SD. Statistical significance was tested by extra sum-of-squares *F* test and fitted to second-order polynomial equation. **c** Tumour growth of individual mice (*n* = 6). Numbers on the right of each graph indicate the number of mice cured per group/number of total mice**. d** Survival after treatment (*n* = 12, pool data of two independent experiments). Statistical significance was tested by the Log-rank test. ***p < 0.001; ****p < 0.0001.

The combination of oleuropein with systemic administration of anti-PD1 antibodies was evaluated in the LCC lung cancer model (Fig. 3a). As controls, groups of mice were treated with saline or with each of the agents singly. Again, oleuropein significantly delayed tumour growth and increased survival. As expected, anti-PD-1 mAb monotherapy failed, confirming the resistance of LCC-derived tumours to PD-1 blockade (Fig. 3c, d). Importantly, oleuropein alone and the combination therapy significantly increased long-term survival (Fig. 3d).

### Oleuropein potentiates PD-1 blockade immunotherapy in colorectal cancer

**Fig. 4:**
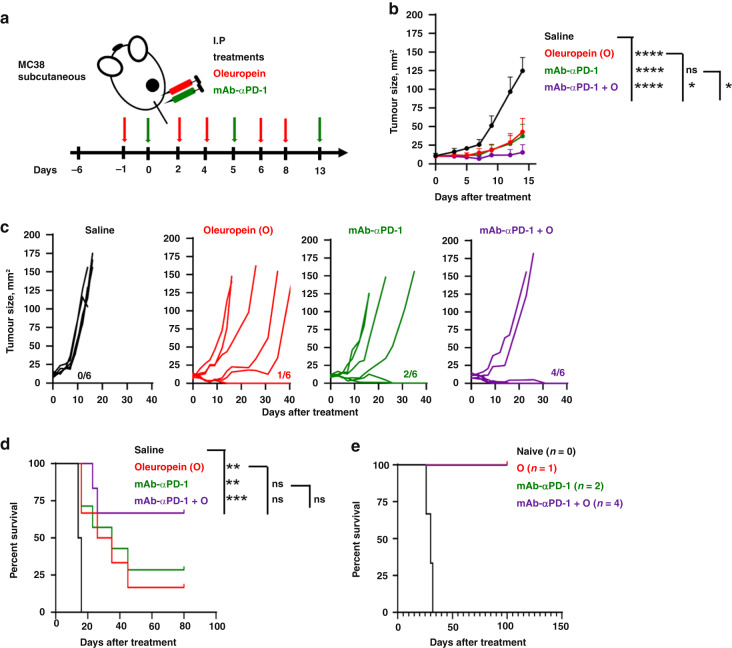
Oleuropein and anti-PD-1 mAb immunotherapy in MC38 colorectal cancer model. **a** Schematic diagram of the experimental setup. Mice bearing subcutaneous MC38 tumours (3–4 mm of average diameter) were treated with 100 µg of anti-PD1 mAb intraperitoneally at the indicated days alone or in combination with oleuropein (O) (300 µg), which was intraperitoneally injected at the indicated timepoints. **b** The graph represents the evolution of mean tumour size following the indicated treatments. Error bars represent SD. **c** Tumour growth in individual mice for the indicated treatment groups. Numbers within the graphs indicate the ratios of cured mice per group/number of total mice. **d** Kaplan–Meier survival plot. Statistical analysis was performed with the Log-rank (Mantel–Cox) test. **e** Kaplan–Meier survival plot for the indicated number of mice that had been previously cured under the indicated treatments, following rechallenge with MC38 tumour cells. *p < 0.05; **p < 0,01; ***p < 0.001; ****p < 0.0001.

The therapeutic efficacy of oleuropein alone and the oleuropein/PD-1 blockade combination were tested in a colorectal cancer model with MC38 cells. Oleuropein was administered intraperitoneally 1 day prior to anti-PD1 mAb injection, followed by four intraperitoneal doses every 2 days. As in previous experiments, mice treated with anti-PD1 mAb received two additional intraperitoneal doses (Fig. 4a).

Oleuropein significantly delayed the growth of MC38 tumours which significantly increased survival with a 16% cure rate (Fig. 4b–d). In this tumour model, anti-PD-1 mAb alone also significantly improved long-term survival with 33% complete regressions. Importantly, oleuropein combined with systemic PD-1 blockade demonstrated a very potent antitumour activity with 66% complete regressions and a significant increase in long-term survival (Fig. 4b, d). Mice with complete remissions remained tumour-free after challenge with MC38 cells (Fig. 4e), demonstrating efficacious memory responses.

### Oleuropein combined with anti-PD-1 delivery within tumours with a SFV vector demonstrates significant therapeutic activities

Intratumour mAb delivery could reduce toxicity by concentrating mAb activity within the TME [25–28]. Hence, a strategy based on intratumour expression of anti-PD-1 mAb in combination with oleuropein was studied. A self-amplifying RNA vector based on SFV was used to express anti-PD-1 (SFV-αPD-1, Fig. S5a). This vector expressed and secreted anti-PD-1 mAb in vitro in BHK and LLC infected cells (Fig. S5b), with the expected specificity and blockade activities (Fig. S5c, d).

**Fig. 5:**
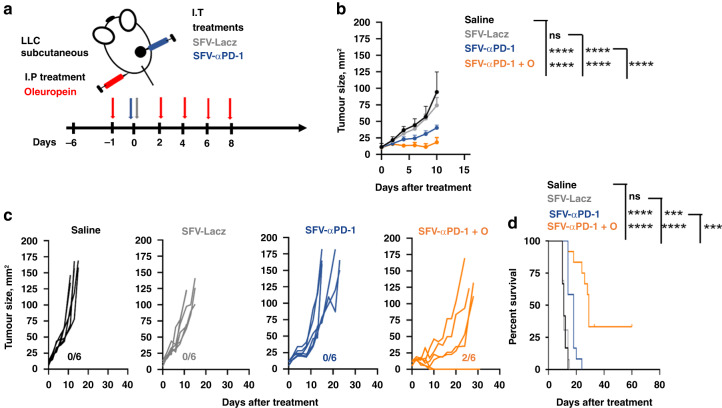
Oleuropein and SFV-αPD1 combination immunotherapy in lung adenocarcinoma LLC tumour model. **a** Schematic diagram of the experimental setup. One million LLC cells were subcutaneously inoculated into groups of C57BL/6 mice. When the average tumour diameter was between 3 and 4 mm, animals received one intratumour dose of 10^9^ VP of SFV-αPD1 or SFV-LacZ. One group of mice received the combination SFV-αPD1/oleuropein (300 µg) at the indicated times (SFV-αPD1 + O). A control group received the same volume of saline. **b** Evolution of mean tumour size following the indicated treatments. Error bars correspond to SD. **c** Tumour growth of individual mice in the indicated treatment groups. Numbers within each graph indicate the number of cured mice/total number of mice. **d** Kaplan–Meier survival plot of mice under the indicated treatments (percent). Survival data was pooled from two independent experiments (*n* = 12). Statistical significance was tested with the Log-rank test. ***p < 0.001; ****p < 0.0001.

Oleuropein was then administered intraperitoneally in combination with a single injection of SFV-αPD-1 within LLC tumours (Fig. 5a). Treatment with SFV-αPD-1 alone reduced tumour growth and significantly increased survival. These results demonstrated that local delivery of anti-PD-1 mAb was efficacious in lung tumours resistant to conventional anti-PD-1 therapy (Fig. 5b–d). Importantly, the oleuropein/SFV-αPD-1 combination demonstrated the most potent therapeutic activity leading to complete regressions and 33% long-term survival (Fig. 5d).

### Oleuropein enhances antitumour responses by altering tumour immune infiltration

**Fig. 6:**
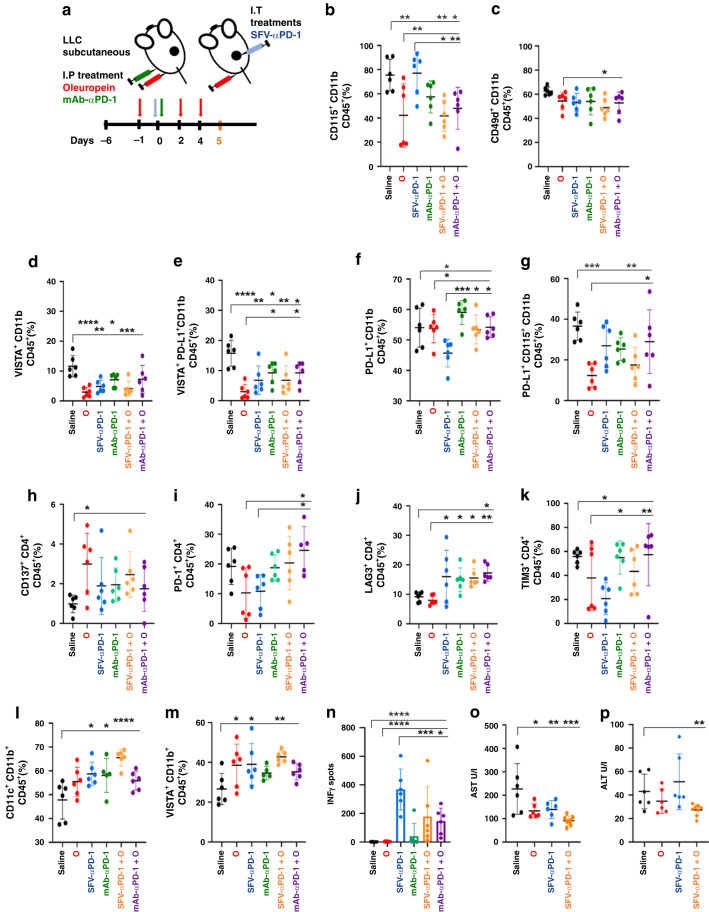
Immune cell populations in tumours and draining lymph nodes (DLNs) following oleuropein and anti-PD-1 therapeutic strategies. **a** Schematic diagram of the experimental setup. Mice were treated as described in (**a**). Analysis of the CD45 immune cells infiltrate in tumours and DLNs. **b**–**g** Percentage of tumour infiltrated myeloid cells (CD11b+CD45+) that express the indicated markers. **h**–**k** Percentage of tumour infiltrating CD4 lymphocytes (CD4+CD45+) that express the indicated markers. **l**, **m** Percentage of DLNs myeloid cells (CD11b+CD45+) that express the indicated markers. **n** IFN-γ-producing cell numbers measured by ELISPOT assay. The graph represents IFN-γ-producing cells/0.7 × 10^6^ T cells. **o**, **p** Transaminase levels (AST and ALT) in serum (*n* = 6 or 8). Data in (**a**–**p**) are expressed as mean ± SD (*n* = 6 mice) and were analysed by one-way ANOVA and Dunnett’s multiple comparisons test. *p < 0.05; **p < 0,01; ***p < 0,001; ****p < 0,0001.

To uncover the mechanisms by which oleuropein exerts antitumour responses in vivo either alone or in combination with PD-1 blockade therapies, mice with LLC-derived tumours were treated with the different therapeutic strategies (Fig. 6a). Tumours and draining lymph nodes (DLNs) were isolated on day 5 post-treatment, and the immune infiltrate was analysed. This time point was chosen because tumour growth delay was evident following treatments (Fig. S5a).

As expected from our previous data, we found differences in myeloid cell composition. Oleuropein significantly decreased CD115+ myeloid cells (macrophages, neutrophils, and MDSCs) (Fig. 6b). The oleuropein/SFV-αPD-1 combination significantly decreased MDSCs (CD49d+CD11b+) (Fig. 6c). Interestingly, the inhibitory checkpoint Vsir (VISTA) was downregulated in all treated groups (Fig. 6d, e). This was particularly evident in CD115+ cells in groups responding better to therapies (oleuropein alone or combined with SFV-αPD-1). PD-L1 expression in myeloid cells was reduced only in mice treated with SFV-αPD-1 (Fig. 6f). However, a closer analysis uncovered a significant reduction in the percentage of cells co-expressing VISTA and PD-L1 (Fig. 6e). No apparent changes in T and NK cell infiltration were observed (Fig. S6c, e), with the exception of a significant increase in CD137+CD4 T cells after oleuropein treatment (Fig. 6h). Mice treated with the oleuropein/αPD-1 mAb combination showed an increase of PD-1+ and LAG3 + CD4 T cells (Fig. 6i, j). In contrast, mice treated with SFV-αPD-1 showed a reduction of PD-1+ and TIM3 + CD4 T cells (Fig. 6i, k), highlighting differences in the mode of action of anti-PD-1 delivery systems. In DLNs, DC infiltration was elevated in all groups especially with the oleuropein/SFV-αPD-1 combination (Fig. 6l). Interestingly, and in contrast to results from the tumour microenvironment, a significant increase in VISTA+ CD11b+ myeloid cells was observed in DLNs for oleuropein, SFV-αPD1, and oleuropein/SFV-αPD-1 treated groups (Fig. 6m). Systemic antitumour T cell responses were evaluated in splenocytes by IFNγ ELISPOT using irradiated LLC cells at a 10:1 ratio (Fig. 6n). SFV-αPD1 treatment was the most potent inducer of systemic T cell responses.

Responses were also studied for SFV-αPD1 or in combination with oleuropein at later times (day 11 post-treatment) (Fig. S7a). Antitumour responses were more evident for all treatment groups at this timepoint (Fig. S7b). These groups also showed a significant decrease in CD49d+CD11b+ MDSCs and CD115+CD11b MDSCs in tumours (Fig. S7c). No changes were found in VISTA and PD-L1 expressing cells (Fig. S7d, f). In tumours, mice treated with SFV-αPD1 showed a significant decrease in CD11b+ cells (Fig. S7g) but an elevation of CD3+ T cells compared with oleuropein (Fig. S7h). All treated mice exhibited significant systemic responses (Fig. S7i). Hepatic and pancreatic toxicities were assessed by quantifying serum transaminase (Fig. 6o, p) and amylase (Fig. S7j). All groups treated with oleuropein, SFV- αPD1 or their combination, showed a significant reduction in AST levels.

## Discussion

PD-1/PD-L1 blockade has demonstrated remarkable clinical outcomes in patients with many tumour types. Nevertheless, these therapies still fail in most cancer patients due to immunosuppression within the TME and systemic immune dysfunctionality [3, 35, 49]. Myeloid cells constitute the major immune constituent of the TME infiltrate, so reprogramming these cell subsets to stimulate anticancer activities could be a promising strategy to overcome treatment resistance. Oleuropein demonstrated potent reprogramming activities over MDSCs and TAMs differentiated ex vivo. Major reorganisations of intracellular pathways were demonstrated by quantitative proteomics, as well as acquisition of immunostimulatory phenotypes leading to enhanced T cell activation capacities. Remarkably, up-regulation of IL-12 in all oleuropein-treated myeloid cell subsets was demonstrated. This is important because IL-12 is one of the most potent anti-cancer cytokines [12, 16, 50–53]. Overall, oleuropein changed differentiation and polarisation of immunosuppressive myeloid cells, including m-MDSCs, g-MDSCs and TAMs. Oleuropein altered cholesterol and fatty acid metabolism by activating LXR/RXR and PPAR-α/ RXR-α pathways, which in macrophages regulate lipid and glucose metabolism as well as inflammatory responses [45, 54]. TREM-1 and cAMP-mediated pathways were also inhibited in all TAMCs. It is important to remark that TREM-1 expression in TAM has been associated to resistance to PD-L1 blockade in hepatocellular carcinoma [55]. cAMP signalling, associated to MDSC and TAM immunosuppressive functions [42, 43], was also down-regulated by oleuropein. VISTA was also downmodulated by oleuropein both in vitro and in vivo in tumour mouse models, an immune checkpoint associated to myeloid cell immunosuppression [48]. Finally, oleuropein polarised in vitro differentiated TAMs towards activated macrophage-like phenotypes. Similar results were observed in myeloid cells isolated from NSCLC patients.

In our study we combined oleuropein with anti-PD-1 mAb immunotherapies. In agreement with previous studies, oleuropein alone exhibited antitumour activities in vitro and in vivo [56–58]. Importantly, oleuropein demonstrated potent reprogramming capacities of myeloid cells, and potent antitumour activities in combination with anti-PD-1 therapies in lung cancer and colon cancer models. One of these corresponded to lung adenocarcinoma LLC “cold” tumours that are intrinsically resistant to PD-1 blockade. In this model, tumour-infiltrating myeloid cells have been shown to be critical determinants of resistance to treatment [8].

Oleuropein was also combined with delivery of anti-PD-1 mAb within the tumours using a SFV-based self-amplifying RNA. This delivery system amplifies immune responses by eliciting type I IFN responses and cancer cell apoptosis [45, 55]. These mechanisms of action could be important for the treatment of refractory tumours to classical immune checkpoint blockade immunotherapies [30, 59]. Indeed, our previous study showed that SFV-αPD-L1 demonstrated superior antitumour activities for the treatment of LLC-derived tumours when compared to conventional anti-PD-L1 mAb therapy [30]. In this study, its combination with oleuropein significantly increased its therapeutic activity. Both oleuropein and anti-PD-1 therapies reduced tumour infiltration by VISTA+ myeloid cells, and increased DC populations within DLNs. This increase was more pronounced with the oleuropein/SFVα-PD-1 combination. These results suggested stimulation of T cell priming by DCs migrating to DLNs to orchestrate a robust antitumour response. These results were supported by the establishment of systemic tumour-specific T cell responses and protective memory responses. Importantly, no toxicity was observed in mice receiving oleuropein, SFV-αPD-1 or their combination, indicating that these therapies could have a good safety profile. In fact, a significant decrease of AST in serum was observed, possibly an indicative of reduced tumour burden.

The combined use of oleuropein and anti-PD-1 mAb administration exhibited a clear synergy for the treatment of MC38-derived tumours. However, such a synergy was not evident in the LLC model when combining oleuropein with systemic administration of anti-PD-1 antibody. However, it is important to remark that synergy was demonstrated when delivering anti-PD-1 antibody within the tumour with an SFV vector. These results indicate that reprogramming of myeloid cells, albeit key to establishing proficient antitumour responses, may not be sufficient for the treatment of poorly immunogenic tumours. While MC38 tumours are good models for immunogenic colorectal cancer, the LLC model is representative of poorly immunogenic lung cancer. Therefore, the use of a cytotoxic SFV vector to deliver mAb causes immunogenic cell death and release of neoantigens that otherwise would remain “hidden” to the immune system. Hence, it is highly likely that reprogramming of the myeloid compartment combined with other strategies that increase tumour immunogenicity will be necessary to improve current immune checkpoint blockade.

Summarising, oleuropein is a potent immunomodulatory agent which reprogrammes immunosuppressive myeloid cells towards immunostimulatory subsets. Oleuropein exhibited potent antitumour capacities and most importantly, enhanced anti-PD-1 therapies in models of lung and colon cancer. Oleuropein improved anti-PD-1 therapies in a tumour model intrinsically resistant to PD-1 blockade, highlighting its potential for cancer treatment. These results in preclinical models lead us to put forward oleuropein as a potential adjuvant for clinical trials.

### Supplementary information

Supplementary figures

Author checklist, ARRIVE

## Data Availability

MS data and search results files were deposited in the Proteome Xchange Consortium via the JPOST partner repository (https://repository.jpostdb.org) with the identifier PXD047616 for ProteomeXchange and JPST002407 for jPOST.
