# Supplementary material for: Oleuropein-driven reprogramming of the myeloid cell compartment to sensitise tumours to PD-1/PD-L1 blockade strategies
Source: Br J Cancer. 2024 Jan 9;130(5):869–79. doi: 10.1038/s41416-023-02561-y (PMC10912768; doi:10.1038/s41416-023-02561-y)
Supplement: Supplementary file 1 — Supplementary figures [file 41416_2023_2561_MOESM1_ESM.pdf]

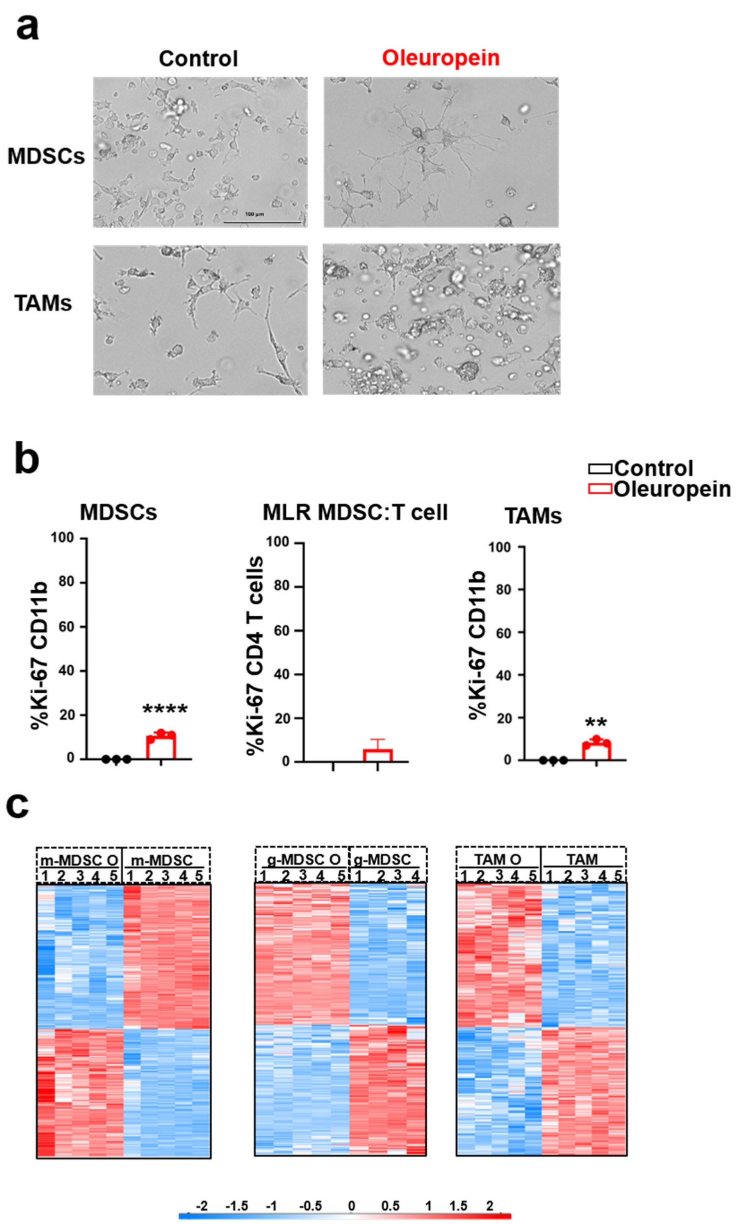

Fig. S1 Phenotype and proteomic profile of myeloid suppressor cell subsets treated with oleuropein. a) Phase contrast micrographs of ex vivo cultures of MDSCs and TAMs treated with oleuropein. b) Bar graphs represent the percentage of CD11b+ MDSCs expressing Ki67, expression within CD4 cells in MLR using MDSC as stimulator cells) and the percentage of CD11b+ TAMs expressing Ki67, treated or untreated with 50 µM oleuropein as indicated. Expressions were normalized to those from control cultures (n=3). Percentages were normalized to those from control cells (n=3 biologically independent cultures). Standard deviations are plotted as error bars. c) Hierarchical unbiased clustering representing the differentially expressed proteins between m-MDSC, g-MDSC, and TAM treated or nor with oleuropein (O) using 4-5 cell culture replicates (two sample test, P<0.01).

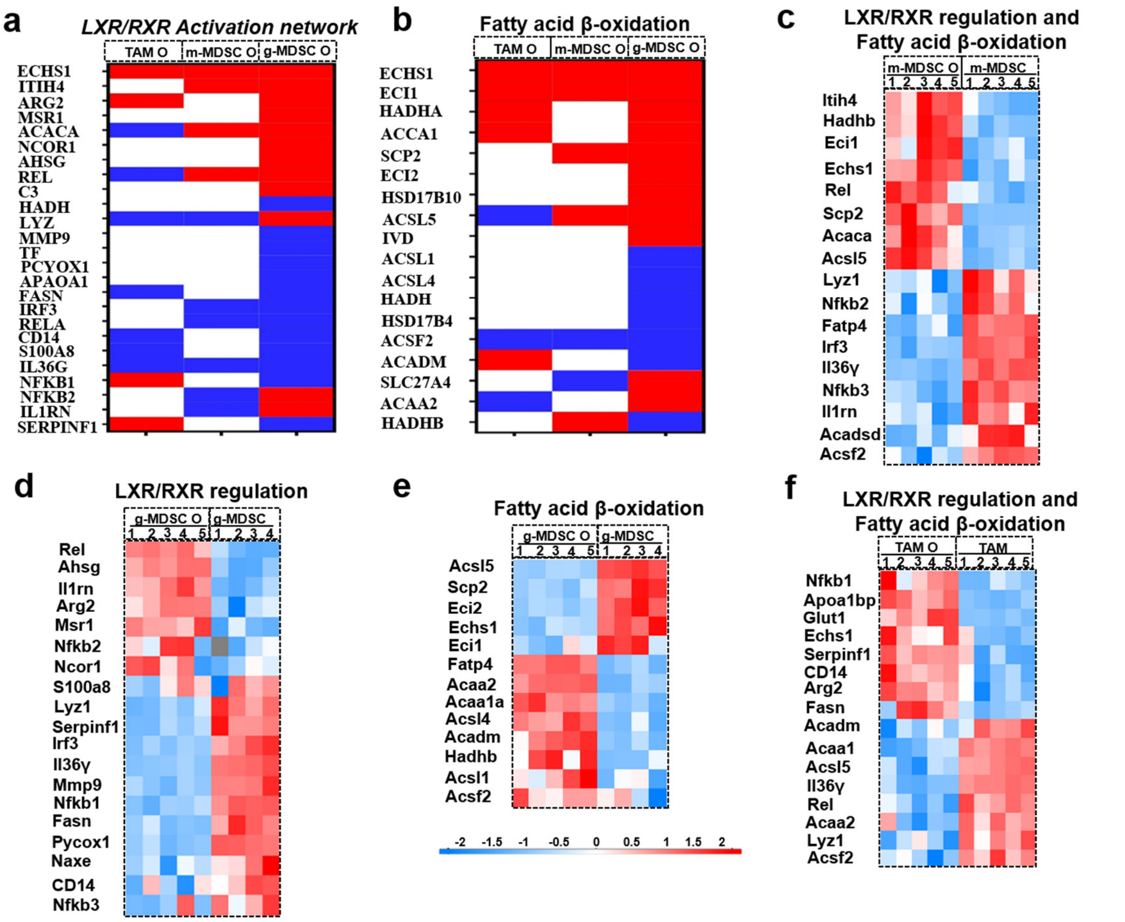

Fig. S2 Pathways regulated by oleuropein in immunosuppressive myeloid cells. (a,b) Canonical pathways enriched in m-MDSC, g-MDSCs, and TAMs treated with oleuropein compared with untreated cells. (**c,d,e**) Hierarchical unbiased clustering representing the differentially expressed proteins in LXR/RXR and Fatty acid β-oxidation pathways between m-MDSC (c), g-MDSC (d,e), and TAM (f) oleuropein-treated (O) or untreated. Proteomic data was obtained from 4-5 cell culture biological replicates (two sample test, $P<0.01$). Data represented in heatmaps according to Z-score of regulators of the indicated canonical pathways. Red and blue, indicate predicted activation or inhibition, respectively, according to z-score.

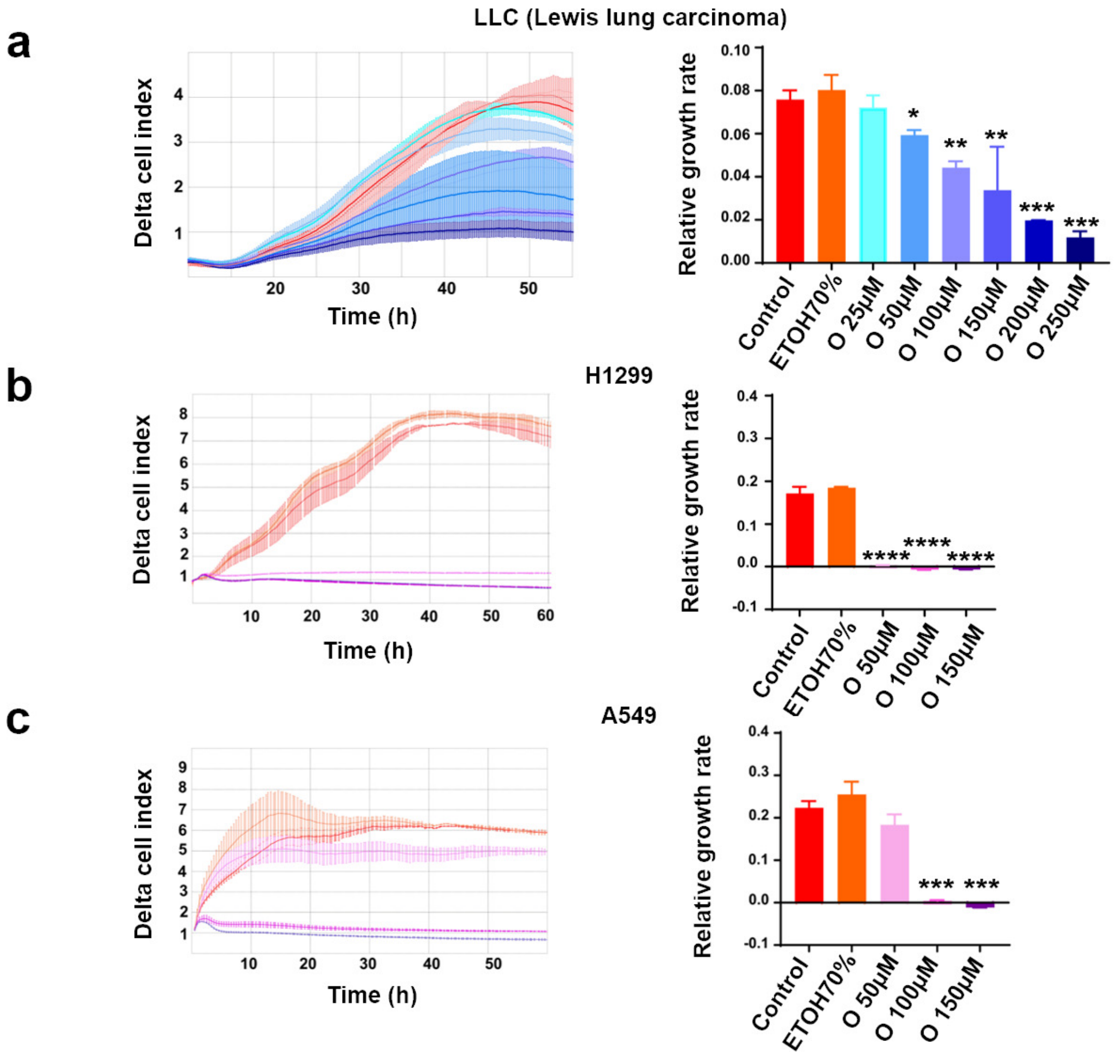

Fig. S3. Oleuropein reduced growth of lung tumor lines in vitro. Growth of Lewis lung carcinoma (LLC), H1299 (b), and A549 (c) cells treated with oleuropein were evaluated by real-time cell analysis (RTCA). Graphs on the left side represent dynamic growth of cells exposed to the indicated concentrations of oleuropein (O), quantified by measuring the delta cell index along time. Graphs on the right side represent growth rates of treated cells relative to non- treated cells. Statistical comparisons were performed by one-way ANOVA followed by a Sidak's multiple comparison test. Non-treated cells (control) and cells cultured with ethanol (etoh) were used as controls.

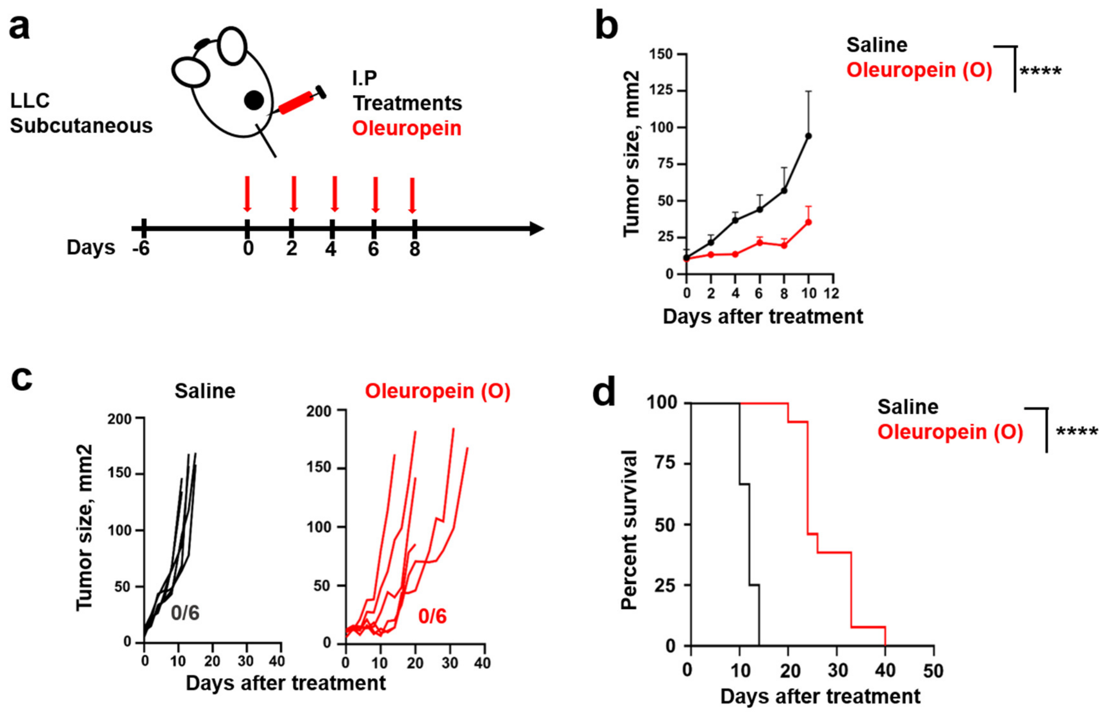

Fig. S4 Evaluation of the antitumor efficacy of oleuropein in LLC tumors. a) Schematic diagram of the in vivo experiment. A total of $1x10^6$ LLC cells were inoculated subcutaneously (s.c.) into the right flank of C57BL/6 mice and approximately six days later (day 0), animals received 300 µg oleuropein (O) given at the indicated times. b) Evolution of tumor size (N=6). Data represent mean tumor volume (mm^2) + SD and differences were analyzed by one way ANOVA at the last time point. c) Tumor growth of individual mice, numbers on the right of each graph indicate the number of mice cured per group/number of total mice. d) Survival (percent) after treatment (N=12, pool of two independent experiments). Survival differences were analyzed by Log-rank (Mantel-Cox) test. ***, $p<0.0001$.

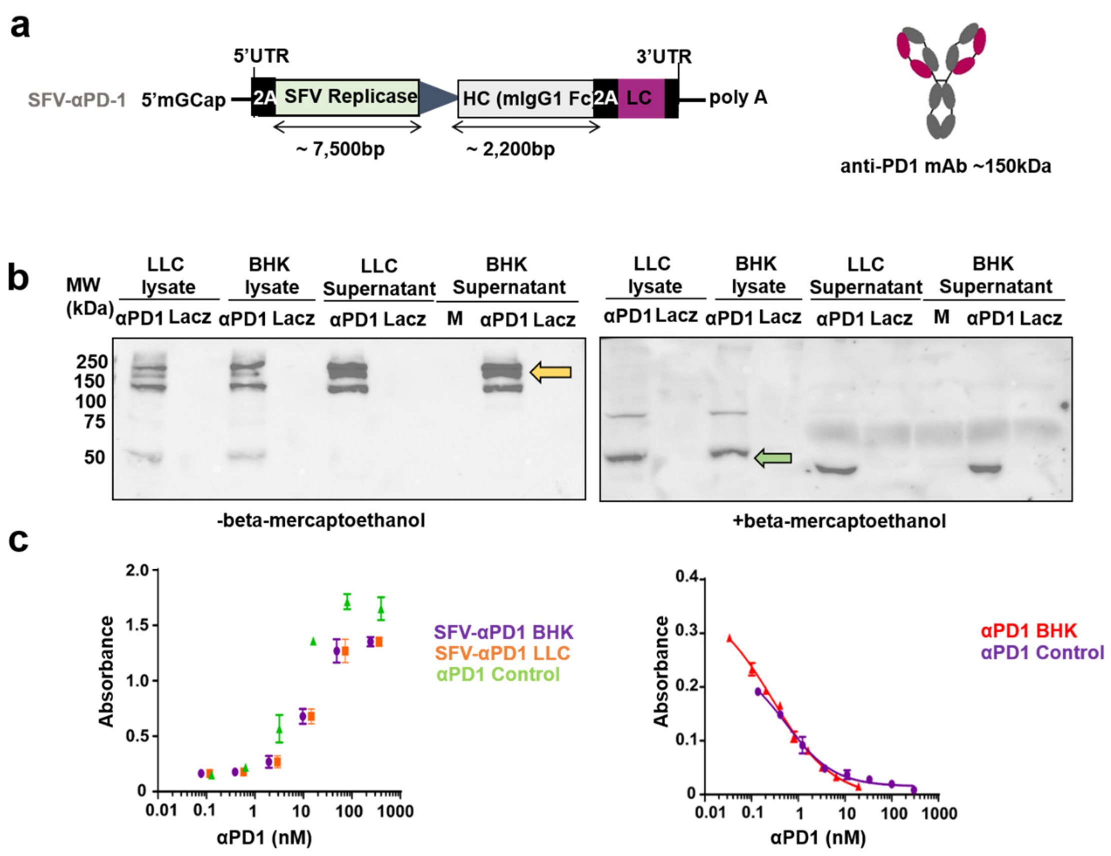

Fig. S5. Characterization of a SFV vector expressing an anti-PD-1 mAb. a) Schematic representation of SFV vector encoding an mAb against mouse PD-1 (anti-PD-1 mAb) in which the heavy chain (HC) and light chain (LC) are linked by foot and mouth disease virus 2A self-protease. On the right side, the mAb with its estimated molecular weight is shown. b-d) BHK and LLC cells were infected with the SFV vectors expressing the indicated transgenes at MOI 25, or mock infected, and supernatants and cell lysates were collected at 48 h and analyzed as indicated. b) Western blot was performed under non-reducing (-β-mercaptoethanol) or reducing (+β-mercaptoethanol) conditions using an anti-mouse IgG peroxidase-conjugated antibody. Yellow and green arrows show the complete mAb and the HC of anti-PD-1 mAb, respectively. M (mock). (c) PD-1 specific binding ELISA using supernatants from BHK (purple) and LLC (orange) infected cells and a commercially available mAb (green) at the indicated mAb concentration. d) PD1/PD-L1 Inhibition curves were performed in a competing binding ELISA using αPD1 mAb from SFV-αPD1 infected BHK cells (red) and a commercially αPD1 mAb (purple). Data represent mean ± SD of the percentage of PD-1/PD-L1 binding, considering wells with no blocking antibody as 100% of binding.

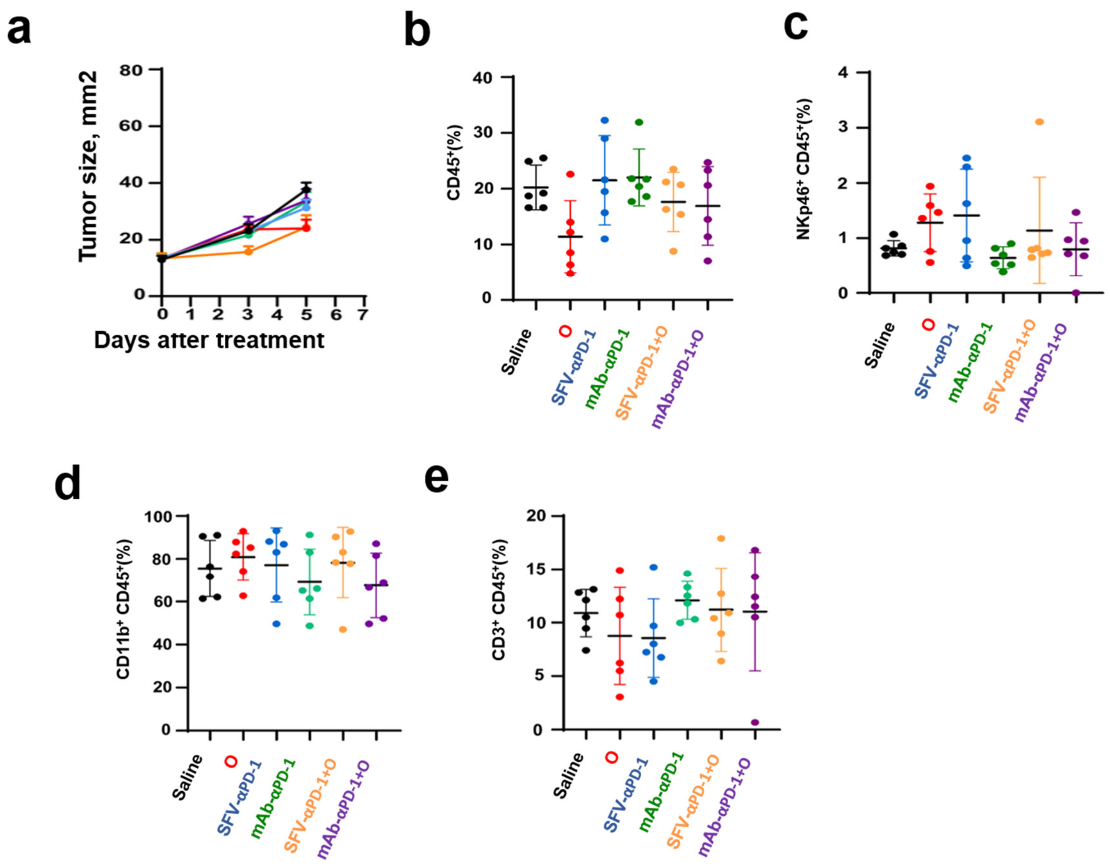

Fig. S6 Analysis of immune cells in LLC tumors treated with oleuropein in combination with anti-PD-1 strategies. a) Evolution of tumor size. Data represent mean tumor size (mm^2) + SEM. b-e) Percentage of cells present in tumors expressing the indicated markers (N=6) and differences were analyzed by one way ANOVA and Dunnett's multiple comparisons test.

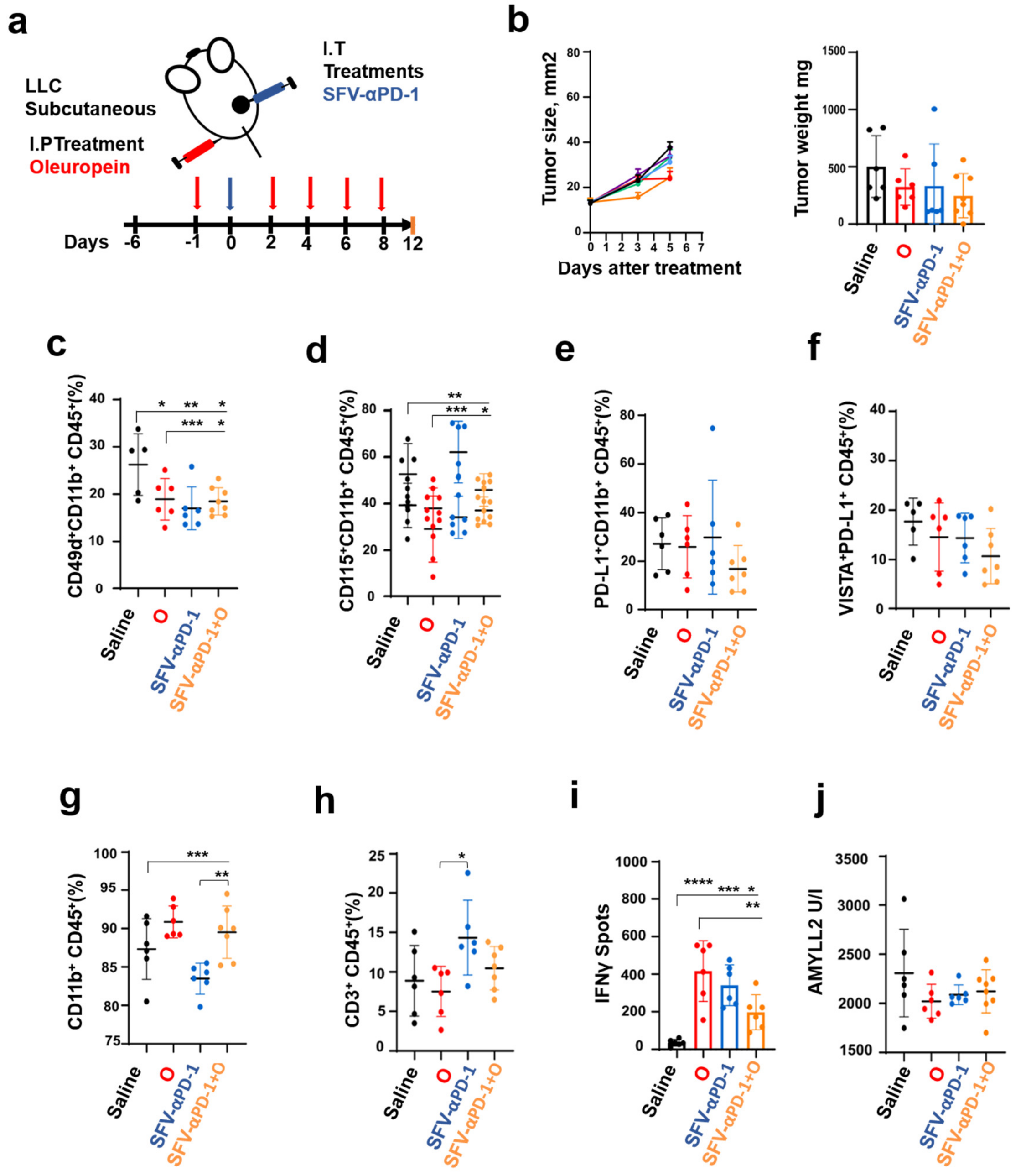

Fig. S7 Analysis of immune cells in LLC tumors treated with oleuropein in combination with SFVαPD-1. a) Schematic diagram of the experiment. Mice having s.c. LLC tumors were treated at day 0 with 10^9 VP of SFV-αPD-1 i.t. (SFV-aPD1), or with the indicated i.p. doses of oleuropein (O) (300ug/mice). Combination group received oleuropein and SFV-αPD-1 (SFVaPD1+O). A control group received the same volume of saline. All mice were sacrificed at day 11 to analyze the immune cell infiltrate. **b**) Evolution of tumor size (left) and tumor weight (right). Data represent mean tumor size (mm^2) + SEM and differences were analyzed by one way ANOVA. **c-g**) Percentage of tumor infiltrated myeloid cells (CD11b+ CD45+) that express the indicated markers. **h**) Percentage of tumor infiltrating CD3 lymphocytes (CD3+ CD45+). i) IFNγ-producing cell numbers measured by ELISPOT assay. The graph represents IFNγ-producing cells/0.7 × 10^6 T cells. **j**) Amylase(AMYL) levels in serum. Data in (c-j) are expressed as mean ± SEM (n = 6 mice) and were analyzed by one way ANOVA and Dunnett's multiple comparisons test. *, p<0.05; **, p< 0,01; ***, p<0,001; ****, p<0,0001.
