# Supplementary material for: Oleuropein-driven reprogramming of the myeloid cell compartment to sensitise tumours to PD-1/PD-L1 blockade strategies
Source: Br J Cancer. 2024 Jan 9;130(5):869–79. doi: 10.1038/s41416-023-02561-y (PMC10912768; doi:10.1038/s41416-023-02561-y)
Supplement: Supplementary file 2 — Author checklist, ARRIVE [file 41416_2023_2561_MOESM2_ESM.pdf]

NOTE: Please save this file locally before filling in the table, DO NOT work on the file within your internet browser as changes will not be saved. Adobe Acrobat Reader (available free here) is recommended for completion.

# The ARRIVE guidelines 2.0: author checklist

## The ARRIVE Essential 10

These items are the basic minimum to include in a manuscript. Without this information, readers and reviewers cannot assess the reliability of the findings.

| Item | | Recommendation | Section/line number, or reason for not reporting |
|---|---|---|---|
| **Study design** | 1 | For each experiment, provide brief details of study design including: | |
| | | a. The groups being compared, including control groups. If no control group has been used, the rationale should be stated. | From 279 to 313 |
| | | b. The experimental unit (e.g. a single animal, litter, or cage of animals). | From 279 to 313 |
| **Sample size** | 2 | a. Specify the exact number of experimental units allocated to each group, and the total number in each experiment. Also indicate the total number of animals used. | Specified in each figure legend |
| | | b. Explain how the sample size was decided. Provide details of any *a priori* sample size calculation, if done. | From 280 to 282 |
| **Inclusion and exclusion criteria** | 3 | a. Describe any criteria used for including and excluding animals (or experimental units) during the experiment, and data points during the analysis. Specify if these criteria were established *a priori*. If no criteria were set, state this explicitly. | No data was excluded |
| | | b. For each experimental group, report any animals, experimental units or data points not included in the analysis and explain why. If there were no exclusions, state so. | No experimental group or data was excluded |
| | | c. For each analysis, report the exact value of *n* in each experimental group. | Specified in each figure legend |
| **Randomisation** | 4 | a. State whether randomisation was used to allocate experimental units to control and treatment groups. If done, provide the method used to generate the randomisation sequence. | From line 280-287 |
| | | b. Describe the strategy used to minimise potential confounders such as the order of treatments and measurements, or animal/cage location. If confounders were not controlled, state this explicitly. | Confounders were not controlled |
| **Blinding** | 5 | Describe who was aware of the group allocation at the different stages of the experiment (during the allocation, the conduct of the experiment, the outcome assessment, and the data analysis). | From line 280-287 |
| **Outcome measures** | 6 | a. Clearly define all outcome measures assessed (e.g. cell death, molecular markers, or behavioural changes). | Explained in detail in methods |
| | | b. For hypothesis-testing studies, specify the primary outcome measure, i.e. the outcome measure that was used to determine the sample size. | Explained in detail in methods |
| **Statistical methods** | 7 | a. Provide details of the statistical methods used for each analysis, including software used. | Explained in detail in methods |
| | | b. Describe any methods used to assess whether the data met the assumptions of the statistical approach, and what was done if the assumptions were not met. | Explained in detail in methods and results |
| **Experimental animals** | 8 | a. Provide species-appropriate details of the animals used, including species, strain and substrain, sex, age or developmental stage, and, if relevant, weight. | Explained in detail in methods |
| | | b. Provide further relevant information on the provenance of animals, health/immune status, genetic modification status, genotype, and any previous procedures. | Explained in detail in methods |
| **Experimental procedures** | 9 | For each experimental group, including controls, describe the procedures in enough detail to allow others to replicate them, including: | |
| | | a. What was done, how it was done and what was used. | Explained in detail in methods |
| | | b. When and how often. | Explained in detail in methods |
| | | c. Where (including detail of any acclimatisation periods). | Explained in detail in methods |
| | | d. Why (provide rationale for procedures). | Explained in detail in methods |
| **Results** | 10 | For each experiment conducted, including independent replications, report: | |
| | | a. Summary/descriptive statistics for each experimental group, with a measure of variability where applicable (e.g. mean and SD, or median and range). | Shown in each figure and specified in figure legends |
| | | b. If applicable, the effect size with a confidence interval. | Explained in detail in methods |
